# Development and Delivery Systems of mRNA Vaccines

**DOI:** 10.3389/fbioe.2021.718753

**Published:** 2021-07-27

**Authors:** Yongjun Liang, Liping Huang, Tiancai Liu

**Affiliations:** ^1^ Key Laboratory of Antibody Engineering of Guangdong Higher Education Institutes, School of Laboratory Medicine and Biotechnology, Southern Medical University, Guangzhou, China; ^2^ Obstetrics and Gynecology Center, Nanfang Hospital, Guangzhou, China

**Keywords:** mRNA vaccine, molecular design, drug delivery, administration, COVID-19 mRNA vaccine

## Abstract

Since the outbreak of SARS-CoV-2, mRNA vaccine development has undergone a tremendous drive within the pharmaceutical field. In recent years, great progress has been made into mRNA vaccine development, especially in individualized tumor vaccines. mRNA vaccines are a promising approach as the production process is simple, safety profiles are better than those of DNA vaccines, and mRNA-encoded antigens are readily expressed in cells. However, mRNA vaccines also possess some inherent limitations. While side effects such as allergy, renal failure, heart failure, and infarction remain a risk, the vaccine mRNA may also be degraded quickly after administration or cause cytokine storms. This is a substantial challenge for mRNA delivery. However, appropriate carriers can avoid degradation and enhance immune responses, effector presentation, biocompatibility and biosafety. To understand the development and research status of mRNA vaccines, this review focuses on analysis of molecular design, delivery systems and clinical trials of mRNA vaccines, thus highlighting the route for wider development and further clinical trials of mRNA vaccines.

## Introduction

With the global spread of the pandemic coronavirus, SARS-CoV-2, during late 2019 and throughout 2020, development of mRNA vaccines has been favored by major research institutions and pharmaceutical giants. Before the outbreak, companies around the world had established various types of mRNA technology research and development platforms, mainly focusing on the prevention of infectious diseases and cancer treatments, such as RNActive of Curevac, mRNA-LNPs of Modena TX and SAM-LNPs. Because of this prior development, especially the pioneer work of Dr. Katalin Kariko, who is currently the senior vice president of BioNTech company in Germany, and participated in the design of several mRNA candidate vaccines of the company. One of them is the bnt162b2 vaccine with an effective rate of 95%. Coronavirus disease (COVID)-19 mRNA vaccines are based on a previously established platform, allowing rapid development and roll-out to save lives all over the world.

However, existing vaccines under development may degrade upon administration or cause cytokine storms (Susanne et al., 2018), among other side effects including allergy, renal failure, heart failure, and infarction. It is therefore essential to find suitable delivery methods for mRNA vaccines to maximize the immunogenic window while minimizing any vaccine-associated risks. Optimized delivery can also enhance the immune response or effector presentation, biocompatibility and biosafety (Susanne et al., 2018).

This review compiles the key delivery system technologies currently available for mRNA vaccines, thus providing references for the future research and development of mRNA vaccines.

## mRNA Vaccines: Where We Began

In the 1990s, Acsadi et al. (1991), Jiao et al. (1992) discovered and demonstrated the therapeutic potential of mRNA. However, due to the instability of mRNA and its easy degradation by RNase, it was considered to be of low applicability at that time. The development of mRNA vaccines and drugs was thus very slow for many years.

However, after the discovery of RNA interference (RNAi) (Tabara et al., 1999) and the Nobel Prize in 2006, the pharmaceutical industry suddenly turned its attention to RNAi treatment, causing a boom in research into RNA as a therapeutic, with inception of many RNAi therapy start-ups. Naturally, large pharmaceutical companies either established partnerships with RNAi companies for joint research and development, offered high prices for powerful mergers and acquisitions, or established internal teams for research and development, leading to expansion of RNA research and consequently paving the way for mRNA vaccine development.

mRNA vaccines are third-generation nucleic acid vaccines developed on the basis of the first-generation attenuated/inactivated vaccines and the second-generation subunit vaccines. At present, nucleic acid vaccines are mainly divided into plasmid DNA vaccines and mRNA vaccines, both of which possess high development potential. The purpose of mRNA vaccines is to transfer RNA to cells for expression and subsequent production of protein antigens, so as to induce an immune response against the antigen, thereby expanding the body’s immune capacity (Thomas and Knut, 2017).

There are two types of mRNA vaccines: nonreplicating mRNA and self-amplifying mRNA. Self-amplifying mRNA not only encodes the target antigen, but also encodes a replicase complex that enables intracellular amplification of the vaccine RNA and enhanced protein expression. Non-replicating mRNA vaccines only encode the target antigen and contain 5' and 3' untranslated regions (UTRs), which provide comprehensive stimulation of the adaptive and innate immunity, namely *in situ* antigen expression and danger signal transmission (Box 1) (Thomas and Knut, 2017; Norbert et al., 2018a).

BOX 1 **Non-replicating mRNA.**

•Can provide comprehensive stimulation of adaptive and innate immunity *via in situ* antigen expression and danger signal transmission.
•Can induce “balanced” immune responses, including humoral and cellular effectors and immune memory.
•Can combine different antigens without increasing the complexity of vaccine formulation.
•Continuous improvement of immune potential can be achieved through repeated vaccination, with no or little immune response to the carrier.
•Heat-stable mRNA vaccines can simplify the transportation and storage of vaccines.

The first step in the mRNA vaccination process is *in vitro* synthesis of the mRNA sequence containing the specific antigen, followed by delivery to the body. This leads to expression of the corresponding antigen protein, which induces humoral and cellular immunity by mimicking virus infection, and ultimately achieves immune protection (Maruggi et al., 2019).

mRNA vaccines maintain the characteristics of DNA vaccines, which can express intracellular antigens while overcoming the shortcomings of low immunogenicity and possible non-specific immunity against the vector, with no risk of integration into host DNA. Compared with DNA vaccines, mRNA vaccines are safer, more effective, and simpler to produce (Norbert et al., 2018b).

However, mRNA vaccines also have limitations (Table 1). The major problem that currently needs to be resolved is the low stability and easy degradation of mRNA molecules. Consequently, the main challenge to development of mRNA vaccines lies in optimizing stability and delivery systems, which can be approached by rational molecular design.

**TABLE 1:** Advantages and disadvantages of mRNA vaccines.

Advantage	Disadvantage
Rapid research and development, simple production process	mRNA is unstable and easily degraded
mRNA vaccines do not require nuclear localization signals and transcription *in vivo*	Strong immunogenicity, triggering unnecessary immune response
No risk of integration into host DNA	Safety is lower than inactivated vaccines
Effectiveness is higher than inactivated vaccines	Effectiveness is lower than DNA vaccines

mRNA vaccines: how we show up.

Pharmaceutical technology for mRNA vaccine has not been mysterious, especially, since the pharmaceutical technology of Pfizer vaccine was accessible for all readers (https://www.nytimes.com/interactive/2021/health/pfizer-coronavirus-vaccine.html?searchResultPosition=2).

The procedure includes 19 steps. At the beginning, plasmids of antigen (proteins, peptides) gene are extracted and they will go into the cells of *E. coli* bacterium. Multiplying bacteria are cultured in LB growth medium (37^o^C) overnight. After that, the multiplying bacteria above will be moved to culture solution and cultured for days of fermentation. Then, *E. coli* bacterium need to be broke up and release the plasmids. The harvested plasmids need to be compared with the samples in order to make sure the coronavirus gene sequence has not changed. After the quality test, the plasmids passing the quality checks need to be cut by enzymes. Enzymes (alkaline phosphatase, ALP or AKP) can linearize circular plasmids and separate the antigen genes (DNA). Linearized antigen genes need to be purified and tested again. Sequentially, linearized DNA is mixed with nucleoside triphosphates (NTPs: ATP, GTP, UTP and CTP) and enzymes (helicases) are used to open the DNA template and transcribe it into mRNA. After purification, the filtered mRNA is tested repeatedly to ensure its accuracy and the accuracy of gene sequence. Finally, the mRNA is encapsulated into liposomes and a vaccine particle is formed.

### Molecular Design of mRNA Vaccines

The mRNA mentioned above would be attacked by the immune system *in vivo*, provided it is natural. For mRNA vaccine formulation, pharmaceutical technology is not in depth. Actually, molecular design of mRNA vaccines plays very important roles, which is patented by pharmaceutical companies. The composition of mRNA includes a 5' cap, 5' UTRs, an open reading frame (ORF), 3' UTRs and a poly(A) tail. mRNA is usually synthesized using linearized plasmid DNA containing the ORF encoding the target protein as a template and is generated by *in vitro* transcription, with the 5' cap and poly(A) tail added at the end of the process. By including the 5' and 3' UTRs, poly(A) tail, 5' cap and nucleoside triphosphate (NTP) on the DNA template for *in vitro* transcription of the mRNA vaccine, the mRNA synthesis elements such as the 5' cap and NTP can be specifically designed to improve the stability and translation efficiency of the mRNA (Norbert et al., 2018b).

### Designing the 5' Cap

**FIGURE 1:**
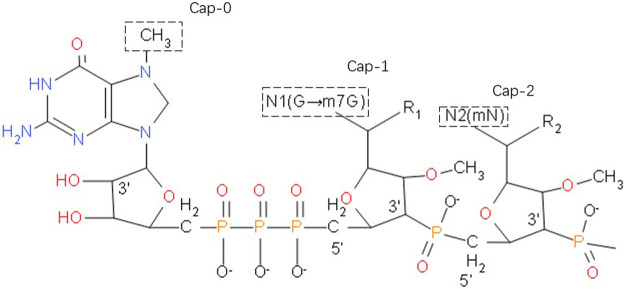
The 5' cap of a eukaryotic mRNA molecule, if a methyl group is added to the 7-position of the end G, the cap with only such a single methyl group is called Cap-0. Plus a methyl in the second base sugar chain 2'-O position, with the above two methyl called Cap-1. When Cap-1 is already present, the 2'-O of the third base sugar chain is also methylated, which is Cap-2.

The stability of mRNA is closely related to the 5' cap, which consists of a positively charged base N7-methylguanosine and a negatively charged 5', 5'-triphosphate bridge (Figure 1). During *in vivo* transcription, eukaryotes utilize three types of caps: Cap-0, m7GppXpYp; Cap-1, m7GpppXmpYp; Cap-2, m7GpppXmpYmp. After addition of a cap, no free terminal phosphate group remains at the 5' end of the mRNA, so it is very stable against alkaline phosphatase (AKP). Moreover, the methyl groups on the two nucleotides behind Cap-1 and Cap-2 block the free 2' OH group on the phosphodiester bond, rendering the molecule very stable against RNase A, RNase T1 and RNase T2.

Alkaline phosphatase (AKP) can catalyze the removal of 5'-phosphate from nucleic acid molecules, thus converting 5'-P of DNA or RNA fragments into 5'-OH terminal. This will improve the efficiency of restructuring and increase volatility of mRNA. The 5'-terminal cap structure of mRNA is a necessary structure for the initiation of mRNA translation, which provides a signal for the recognition of mRNA by ribosomes, assists the binding of ribosomes and mRNA, and makes translation start from AUG. Cap structure can protect mRNA from 5'→ 3' exonuclease attack. Samarina et al. found phosphatase resistent, blocked 5'-termini by different methods including treatment by alkaline phosphatase (Samarina et al., 1979).

However, in addition to the methylation of nucleotides, the hydrogen bonds within the phosphodiester bonds may also be methylated. Furthermore, the cap polymer can be inversely combined to form isomers. Both of these scenarios will cause downstream processes to be affected. To solve these problems, various cap analogs have been developed, called anti-reverse cap analogs (ARCAs). ARCAs are modified at the C2 or C3 positions to ensure that the methyl group replaces the OH at the correct position during transcription. For example, 3'-O-Me-m7G (5)-pppG (5), in addition to the guanosine N7-methyl substituent, also has a methyl group on the 3'-OH of m7G ribose. Compared with the traditional cap structure, ARCA-capped mRNA has higher translation efficiency (Zheng-Hong et al., 2002). m27, 2'−OGppSpG (β-S-ARCA) phosphorylates thiocaps can greatly improve the translation efficiency and enhance the stability of RNA of dendritic cells, of which the D1 diastereomer effects most. This may be related to the resistance of ARCA to DCP2’s uncapping effect (Kuhn et al., 2010). Cap analogues modified by locked nucleic acid (LNA) is one of the substrates of T7 RNA polymerase. The mRNA transcribed by cap analogues has poly 1) tail, which can be used for *in vitro* translation. Studies have shown that the RNA of cap modified by 5'–LNA is 1.61 times more stable than that of cap modified by 5'–standard4, 1.28 times more stable than that of cap modified by Arca, and 4.23 times more stable than that of cap unmodified. The translation efficiency of RNA modified by M [7 (LNA)] g (5') PPP (5') g3cap analogue was the highest, about 3.2 times of that of standard CAPM (7) g (5') PPP (5') G4 (R, Kore et al., 2009).

### Designing the 5' UTR

**FIGURE 2:**
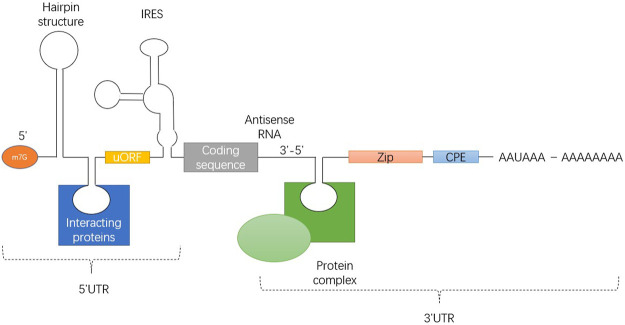
Universal structure of eukaryotic mRNA, showing the structure of the 5' and 3' UTRs.

UTR includes stem loop structure, upstream initiation codon and open reading frame (ORF), internal ribosome entry site (IRES) and various cis elements that can be bound by RNA binding proteins. Figure 2 illustrates 7-methyl-guanosine cap (m7G), hairpin-like secondary structures (hairpin), upstream open reading frame (uORF), internal ribosome entry site (IRES), Zip code, cytoplasmic polyadenylation element (CPE) and polyadenylation signal AAUAAA from5' side to 3' side.

The translation efficiency of mRNA is affected by the structural characteristics of the 5' UTR (Figure 2). Regulation of translation initiation is a central control point in animal cells. UTRs are usually not encapsulated by ribosomes, and they may interact more easily with regulatory factors. The 5' UTR sequence can determine which initiation pathway is used to bring the ribosome to the initiation codon, how efficiently initiation occurs, and which initiation site is selected.

Translation initiation requires 43S pre initiation complex to scan 5' UTR reaches the initiator codon, and the 5' UTR with high GC content will lead to low startup rate and scanning efficiency. Therefore, the 5' UTR which is rich in negative folding free energy and GC is often used to predict the parameter 5' UTR of RNA secondary structure. Studies have shown that the complex secondary structure of 5' UTR which is rich in GC is related to inhibition of translation in guanine decarboxylase 36 mRNA (Leppek et al., 2018).

Studies have shown that 5' UTRs should not contain upstream ORFs, thus avoiding erroneous translation initiation and replacement reading frames (Gray and Marvin, 1998). It has been reported in the literature that 5' UTRs must be short and loose because the stable secondary structure prevents small molecule ribosomes from binding to the initial coding element. Studies have shown that as the number of linkers increases, the translation efficiency decreases, supporting the view that excessive secondary structure at the 5' end of eukaryotic mRNA hinders translation (Pelletier and Sonenberg, 1985). The 5’UTR of the transcript of capsid mRNA is composed of exons. Exon 2 and exon 3 affect mRNA abundance most, and exon 4 regulates the translation of capsid protein.(Xiaoqian et al., 2018).

### Designing the 3' UTR

The 3' UTR is a concentrated area of mRNA instability factors, among which A + U-rich elements (AREs) (Murray and Schoenberg, 2007) and GU-rich elements (GREs) (Louis and Bohjanen, 2011) are common factors. Therefore, these sequences should be avoided when synthesizing mRNA.

In addition to avoiding ARE and GRE sequences, the introduction of stabilizing elements can also improve the stability of mRNA and prolong its half-life. For example, BioNTech uses two consecutive copies of the human *β*-globin 3' UTR in its patent (now called 2 hBg; previously also called 2βgUTR) to contribute to higher transcript stability and translation efficiency (WO 2017/059902 Al; BioNTech RNA Pharmaceuticals GmbH, Mainz, Germany). Studies have shown that the 3' UTR of human *α* and *β* globulin can enhance the stability and translation efficiency of mRNA, and the 3' UTR of two human *β*-globulins arranged head to tail can increase the stability of mRNA (Mehrije et al., 2015).

### Nucleoside Analog Design

Nucleosides are the components of DNA and RNA, usually containing bases and ribose. Structure-modified nucleoside analogs (NAs) can interfere with the biological process of DNA and RNA synthesis from nucleosides. Therefore, nucleoside analogs are often used as tool molecules in the field of life science research. Uracil analogs are the most common nucleoside analogs used in mRNA vaccines. The immunogenicity and stability of this kind of mRNA containing nucleoside analogues have been improved, with vaccines under development for a range of viral infections using this approach.

DNA and RNA can stimulate mammalian immune system by activating Toll like receptors (TLRs). However, DNA containing methylated CpG motif does not have stimulating ability. Studies have found that RNA signals are transmitted through human Toll-like receptors (TLR)3, TLR7 and TLR8, but the addition of modified nucleosides m5C, m6A, m5U, s2U or pseudouridine inactivates this signaling (Karikó et al., 2005). Using methyl pseudouridine instead of uridine-5'-triphosphate (UTP) to synthesize a modified HA mRNA-LNP influenza vaccine can elicit a lasting specific antibody response against the HA spike in mice, rabbits and ferrets (Pardi et al., 2018). Similarly, this approach has been used to generate an mRNA-LNP vaccine against HIV-1, specific antibodies were detected in all immunized animals s after both kinds of immunization (Norbert et al., 2019).

Nelson and colleagues found that a nucleoside-modified mRNA vaccine against human cytomegalovirus glycoprotein B (gB) caused a more durable and extensive antibody response compared with gB protein immunization with MF59 adjuvant (Nelson et al., 2020a).

### Designing the 3' poly(A) Tail

3' poly(A) tail is the 3' end of eukaryote mRNA. Most eukaryotes have poly(A) tails composed of dozens to hundreds of a bases at the 3' end of mRNA.

The addition of 3' poly(A) tail to eukaryotic mRNA is a default process. Almost all eukaryotic mRNA is modified after transcription to obtain poly(A) tail with 250–300 A bases. However, the length of 3' poly(A) tails was highly regulated in the nucleus and cytoplasm. Different lengths of 3' poly(A) tails help to regulate the stability, transport and translation of mature mRNA. Once the mRNA reaches the cytoplasm, the poly 1) tail cooperates with the 5' cap to form a stable closed loop structure with the eIF4F complex and promote translation initiation. The length of 3' poly(A) tail determines the degree of mRNA translation. The shortening and prolonging of 3' poly(A) tail can well reflect the regulation of gene expression by 3' poly(A) tail in time and space.

The 3' poly(A) tail cooperates with the 5' cap, internal ribosome entry site and various other determinants to regulate mRNA stability and translation efficiency. In most eukaryotic cells, 3' poly(A) is one of the most important structures for mRNA stability; most mRNA degradation starts at the 5' Cap and the 3' poly(A) tail.

Currently, there are several different approaches to the poly(A) tail used in different mRNA vaccines. In the mRNA influenza vaccine studied by Pardi et al. (2018), a poly(A) tail with a length of 101 nt was used, while BioNTech disclosed in a published patent that the mRNA stability and translation efficiency of the 120 nt 3' poly(A) tail was higher than that of the 16 nt, 42 nt, and 67 nt mRNAs (patent WO 2017/059902 Al, BioNTech RNA Pharmaceuticals GmbH).

### mRNA Vaccine Delivery Systems

Efficient entry of mRNA vaccines into human cells is a very challenging process. As an exogenous nucleic acid, naked mRNA is easily recognized by the immune system and rapidly degraded by nucleases after entering the body. Therefore, the pharmacological effects of using naked mRNA as a vaccine are greatly reduced. To improve the immune efficiency of mRNA vaccines, special delivery systems are required to protect administered mRNA from nucleases and allow delivery into cells.

For mRNA vaccines to work, it is vital that they are successfully delivered into target cells in the human body. Electroporation is often used to transfer mRNA into *ex vivo* dendritic cells (DCs) before autologous transfusion into patients. This method has proven effective in cancer immunotherapy (Axel et al., 2002).

Although mRNA vaccines do not enter the nucleus, they need to enter the cytoplasm and then be translated into target protein. So to reach the cytoplasm, mRNA needs to pass through the negatively charged phospholipid bilayer of the cell membrane. Only molecules which are smaller than 1,000 Da can enter cells through passive diffusion, while naked mRNA has a relatively large molecular weight and so requires a carrier to enter the cell. Less than 1 μm particle can be directly internalized by non phagocytic eukaryotic cells, such as pathogens and liposomes. When the particle size is less than 200 nm, the microspheres need to be coated with gridding protein, while larger particles of 500 nm are dependent on caveolae-mediated internalization (Joanna et al., 2004).

Delivery systems for mRNA vaccines can be divided into viral- and non-viral vector delivery systems. This review mainly discusses non-viral vectors, which can be further subdivided into lipid or lipid materials and polymer delivery systems. The hybrid system of lipid and polymer are also advantage for macromolecule (e.g. mRNA) delivery (Xue et al., 2017). Furthermore, nanoparticle vaccines are becoming increasingly popular.

## Lipids or Lipid Materials Used to Deliver mRNA

### Liposome Complexes

Cationic liposomes were the first liposome delivery materials used in mRNA vaccines. Liposomes are spherical vesicles composed of a single or multiple layers of phospholipids. The vesicle has an aqueous core containing the target gene, and is usually prepared using materials containing polar head groups and non-polar tails. The hydrophobic and hydrophilic interactions between these groups stimulate vesicle formation. Positively charged cationic lipids can aggregate with negatively charged mRNA through electrostatic interactions to form a multi-layer cystic complex called a lipoplex (LP). The mRNA encapsulated in the LP is not easily accessed by RNase and so can be delivered without degradation. However, because cationic lipids are also positively charged under physiological conditions, they are likely to interact with other negatively charged molecules in biological fluids, and are also easily captured by immune cells, resulting in poor delivery effects. On this basis, pH-responsive cationic lipids are screened and made into various forms of mRNA delivery vehicles. Charlotte et al. (2013) demonstrated that a complex antigen T cell reaction can be produced when the mRNA encoding HIV gag protein was bound to cationic lipid DOTAP/DOPE. It can induce the specific killing of cells and the humoral response. In addition, they also found that the mRNA encoded by DOTAP/DOPE complex antigen has the characteristics of immune activation, which is characterized by the IFN-I and the pre-inflammatory monocytes. They also demonstrated that the antigen-specific immune response and expression of antigen-encoding mRNA complexed with DOTAP/DOPE were inhibited by type I IFN.

### Liposome Nanoparticles

Liposome nanoparticles (LNPs) are currently the most advanced delivery system for mRNA vaccines. Originally used for the delivery of siRNA, LNPs have been proven to be safe and effective (Ariful et al., 2015). LNPs are stable particles composed of a lipid bilayer shell of cationic lipids, auxiliary lipids, cholesterol and polyethylene glycol (PEG) encompassing an aqueous core (Kuntsche et al., 2011), in which they can carry an mRNA payload.

Dr. Pieter Cullis is a pioneer in field of liposomal RNA delivery. Pieter Cullis et al. found that liposome nanoparticles encapsulated antigen can enhance the immune efficacy and induce stronger immune response (Jong et al., 2007). This preliminary revealed the advantages of liposome nanoparticles encapsulation in immunology.

Norbert et al. (2015) studied the effect of the route of administration on the delivery of mRNA by LNPs. They found that when mRNA-loaded LNPs were administered at doses of 0.005–0.25 mg/kg, subcutaneous, intramuscular and intradermal injections mediated the local expression of the protein encoded by the mRNA. They further demonstrated that intravenous and intraperitoneal injections also mediated systemic mRNA transport, enabling target proteins to be expressed in the liver for up to 4 days.

Maja et al. (2018) evaluated the safety of LNP packaging of human erythropoietin (hEPO) mRNA in monkeys and rats. following intravenous injection of 0.3 mg/kg mRNA LNP, hEPO levels were highest at 6 h post-infusion and exceeded the expected effective exposure 100-fold at the maximal dose. Active hEPO protein was detected in both rats and monkeys, indicated by significant increase in erythrocyte mass. Some increase in white blood cell count, changes in coagulation parameters, liver injury and release of IFN-γ-induced protein were observed in rats, while in monkeys, splenic necrosis, lymphocyte depletion and mild, reversible complement activation were observed. These pro-inflammatory responses may be minimized by reducing the dose or the frequency of administration.

mRNA can be encapsulated with lipid nanoparticles to induce high levels of GCB and TFH cells, produce antigen-specific CD4^+^ T cell response, and produce effective neutralizing antibody response. The TFH cells and GCB cells is related to the production of antibodies which are high-affinity and long-lived, and this can provide a long-term protection (Norbert et al., 2018b).

### Polymers for mRNA Delivery

Since polylysine (PLL) was reported as the first cationic polymer non-viral vector to successfully transfect plasmid DNA in 1987 (Wu and Wu, 1987), polymer non-viral vectors such as spermine, polyethyleneimine, chitosan, and polyurethane have become readily available. At present, common polymer delivery systems are poly-amido-amine (PAA), poly-beta amino-esters (PBAEs) and polyethylenimine (PEI).

PEI was originally used as a non-viral vector to deliver DNA into the mouse brain, demonstrating the high efficiency of this polymer as a delivery vector (Boussif et al., 1995), while also enhancing siRNA or DNA transfection efficiency (Jere et al., 2009a). Although PEI has unique advantages as a gene delivery system, its severe cytotoxicity limits its application (Jere et al., 2009b). As the molecular weight of PEI increases, the transfection efficiency increases, as does the toxicity. To overcome such limitations, various modifications to PEI have been investigated, such as modifications with polysaccharides and polyethylene glycol to improve biocompatibility and transfection efficiency, and wrapping PEI with anionic liposomes or PEI/nucleic acid polymers encapsulated by neutral liposomes to reduce non-specific adhesion. In addition, preparation of low molecular weight PEI derivatives can reduce cytotoxicity while improving transfection efficiency (Jere et al., 2009b).

Zhao et al. (2016) prepared a self-assembled cationic nanomicelle using PEI stearic acid (PSA) copolymer as a carrier to introduce the HIV-1 gag gene into dendritic cells and BALB/c mice. Compared with PEI-2k polymer (PEI with molecular weight of 2000), the cellular uptake efficiency of PSA nanoparticles was higher.

### Other Nanoparticle Materials for mRNA Delivery

Wang et al. (2020) designed a ferritin nanoparticle vaccine to deliver PreS1 to specific bone marrow cells. The vaccine can induce strong and persistent anti PreS1 response and effectively eliminate the virus in mice with hepatitis B.

Bahmani et al. (2021) investigated the antitumor effect of resiquimod (R848), a delivery of TLR agonist, mediated by platelet membrane-coated nanoparticles (PNP). PNP can enhance the interaction between R848 and tumor, and maximize the activity of R848. Intratumoral injection of pnp-r848 can significantly enhance the local immune activity, make the tumor completely subside, and inhibit the recurrence of tumor. In addition, they also found that nanoparticles carrying agonists have the ability to delay tumor growth and inhibit metastasis. It is noticeable that using biomimetic nanocarriers to deliver immune stimulation by local delivery has the potential to enhance biocompatibility and natural targeting affinity.

### Different Routes of Administration

Liposome complexes are usually injected intravenously. Hess et al. (2006) used an ovalbumin (OVA)-expressing tumor model in C57BL/6 mice followed by intravenous injection of OVA mRNA in a 1,2-dioleoyl-3trimethylammonium-propane/1,2-dioleoyl-sn-glycero-3-phosphoethanolamine (DOTAP/DOPE) vector, one kind of modified cationic liposomes. resulting in inhibition of tumor growth.

Various routes, including tracheal inhalation, intravenous, intraperitoneal, and intramuscular injections can mediate systemic mRNA transport and expression. Intravenous injection is a systemic administration, which can deliver mRNA vaccine to target area through blood circulation and play a role quickly. Intramuscular injection can deliver mRNA to muscle tissue, which can be administered in larger doses. Liposomal nasal administration is appropriate and can reach local lymph tissue quickly. Of these, intravenous injection has the best effect, enabling mRNA gene translation proteins to be expressed in the liver for up to 4 days (Norbert et al., 2015).

## mRNA Vaccines: Where We Are Now

### mRNA Vaccines That Have Completed Clinical Trials

Prior to the outbreak of the SARS-CoV-2 pandemic in late 2019, mRNA clinical trials had previously focused on malignant melanoma, prostate cancer, acute myeloid leukemia, HIV-1 and other cancers. The clinicaltrials.gov database includes details of all registered clinical trials of mRNA vaccines, most of which are in clinical phase I/II. Completed clinical studies of mRNA vaccines include eight studies regarding mRNA transfection of mRNA into patients’ DCs followed by autologous transfusion via intradermal, intravenous or lymph node injection, and two studies investigating intradermal injection of mRNA. One of the studies investigate intradermal injection of mRNA is modified mRNA encoding VEGF-A (Gan et al., 2019).

Gandhi et al. (2016) and his team transfected autologous DC cells with mRNA encoding HIV-1 Gag and Nef. Fifteen subjects were randomized to receive vaccine or mock transfected DC placebo. The results showed that there was no difference in ELISPOT response to HIV-1 gag or Nef between the two groups. Gandhi et al. suggested that the enhancement of response to HIV-1 antigen and KLH antigen was temporary. Therefore, Dendritic cell vaccines should be modified. If dendritic cell vaccination can cause a long-lasting and stronger immune response, this strategy can become a therapeutic vaccine for HIV-1.

For melanoma, (Kyte et al. (2007) developed a personalized vaccine of melanoma based on dendritic cells transfected with mRNA of autologous tumor. In the inoculated samples, 39 T cell clones were generated, and they all responded to the stimulation of DC transfected with mRNA. Among them, 12 clones responded to the simulated transfected DC. The results showed that 10/11 clones had different TCR. This finding indicates that the cytokine response after cancer vaccination is more complex than the classical Th1/Th2 dichotomy.

### COVID-19 mRNA Vaccines

After the outbreak of COVID-19, the world’s major pharmaceutical companies and scientific research institutions have devoted themselves to the field of vaccine research and development. Currently, there are more than 200 post-selection new coronavirus vaccines, and 17 of them have entered the clinical trials stage. Among them, mRNA vaccines are attracting attention, with the global development of mRNA vaccines mainly concentrated in three companies: BioNTech (Mainz, Germany), CureVac (Tubingen, Germany) and Moderna (Cambridge, MA), in cooperation with major pharmaceutical companies. New coronavirus vaccines based on mRNA technology that have entered clinical stages are listed in Table 2.

**TABLE 2:** mRNA COVID-19 vaccines undergoing clinical testing.

Institutions	Vaccine name	Progress	Delivery system and mRNA coding
Institute of Military Medicine, Chinese Academy of Military Sciences	ARCoVax	WHO has evaluated the efficacy and safety data of phase III clinical trial of COVIV-02NCT04510207	Lipid nanoparticles encapsulate SARS-CoV-2 receptor- binding domain (RBD) Zhang et al. (2020)
Pfizer and BioNTech	BNT162b2	Phase I/II clinical data show good performance in safety, tolerability and immunogenicity. Phase III clinical trials has been launched. BNT162B2 has now been granted emergency authorization for administration in several countries, and has been rolled out throughout 2021	Lipid nanoparticles encapsulate SARS-CoV-2 trimeric Spike glycoprotein receptor- binding domain (RBD) Mulligan et al. (2020b)
Moderna and the National Institute of Allergy and Infectious Diseases (NIAID)	mRNA-1273	Early trial data showed that the vaccine produced neutralizing antibodies against the new coronavirus in at least 8 volunteers, but the phase III clinical trial was postponed	Liposome nanoparticles encapsulate modified SARS-CoV-2 Spike glycoprotein Jackson et al. (2020)

After Pfizer/BioNTech and Moderna’s COVID-19 vaccine candidates BNT162b2 and mRNA-1273 entered the marketing application stage in Europe (EMA) and Canada, respectively (October 27, 2020), the British Medicines and Healthcare Products Regulatory Agency (MHRA) also launched a rolling review procedure for mRNA-1273.

Pfizer/BioNTech vaccine has been used in many countries and regions, such as European Union, France, Australia et al. According to Pfizer and BioNTech, the phase three trial of Pfizer/BioNTech vaccine involved 43,000 volunteers from approximately 150 clinical trial sites in the United States, Germany, Turkey, South Africa, Brazil and Argentina. The overall effective rate was 95%, and the effective rate of people over 65 years old was 94%.

### mRNA Vaccines: Where We Are Going

At present, the popularity of mRNA technology is ever increasing. mRNA approaches have two very attractive applications, they are the use of mRNA vaccines for cancer and viral diseases, and for the treatment of non-targeted gene diseases. The mRNA vaccine production process is simple, rapid, and low cost; such vaccines have an adjuvant effect to activate the immune response; they do not require transcription in the nucleus and so have no risk of integration into the host genome; they can be used as an endogenous anti stress substance and presentation of encoded peptides are promoted by MHCI molecules, resulting in activation of the cytotoxic T lymphocyte response to kill tumor cells. On the basis of the development of cancer gene sequencing and antigen neo-epitope discovery technology, mRNA vaccines have become the best choice for personalized tumor vaccines.

The COVID-19 pandemic has provided a unique opportunity to accelerate the development of mRNA vaccine technology. The COVID-19 vaccine mRNA1273 jointly developed by Moderna and the National Institute of Allergy and Infectious Disease (NIAID) was the world’s first COVID-19 vaccine to undergo clinical trials, taking only 63 days from the release of the SARS-CoV-2 genome sequence to clinical phase I, highlighting the potential of this vaccine technology for rapid, dynamic and versatile development against emerging diseases.

In the past two years, dozens of mRNA vaccines for prevention of infectious diseases and cancer treatment have released preclinical and clinical trial research reports, confirming the reliability and effectiveness of the existing mRNA vaccine technology platform. The rapid advancement of COVID-19 mRNA vaccines to the marketplace is partly due to the outbreak and severity of the SARS-CoV-2 pandemic, and such developments have been based on previous candidate products in this field. The Pfizer/BioNTech vaccine is now approved for emergency use by the FDA and being administered in several countries and regions above. The vaccine of the Institute of Military Medicine, Chinese Academy of Military Sciences has been widely used in China and some other countries.

Despite many vaccine candidates remaining in the pre-clinical and early clinical phase I–II stages, this has not dampened enthusiasm for research and development in the field, with ever increasing numbers of companies and institutions entering the arena. Although the fastest-developing mRNA vaccine (mRNA1647 by Moderna) for preventing cytomegalovirus infection remains in clinical stage II, fully approved commercial mRNA vaccines are rapidly approaching the market, with companies forging deals for development and distribution, such as the agreement signed between. According to Ugur Sahin, the founder and CEO of BioNTech, Fosun Pharma and BioNTech, authorizing exclusive development and commercialization of a novel coronavirus vaccine based on the BioNTech proprietary mRNA technology platform in China.

There are still some challenges to mRNA vaccines. Despite the rapid recent advances in mRNA vaccine technology, more efficient activation of the immune response, particularly against tumor cells, remains challenging. In addition, development of optimal delivery systems to protect the mRNA payload from degradation within the complex internal milieu is critical. In terms of quality control, detection of residual template DNA and incomplete mRNA is also difficult. However, the advantages of the mRNA vaccine approach are numerous and, in addition to being used as a vaccine, mRNA can also be used as a protein supplement or replacement therapy to treat other diseases, driving the necessary research to overcome the current obstacles and limitations with the ultimate goal of developing an ideal form of medicine.

## Limitations

There may be some possible limitations in this study. There are more ways to improve the stability and translation efficiency of mRNA. Here, we provide a broadly vision in molecular design and delivery system in order to give readers a way to design mRNA vaccine.

It is not in depth for mRNA vaccine formulation in this review. In the section of *Preparation of mRNA vaccine*, we talk about an extensive method of making mRNA vaccine based on a small sample size. Real mRNA vaccine production needs more details and experimental data.
