# Corrigendum: Development and Delivery Systems of mRNA Vaccines

**DOI:** 10.3389/fbioe.2021.766764

**Published:** 2021-09-17

**Authors:** Yongjun Liang, Liping Huang, Tiancai Liu

**Affiliations:** ^1^ Key Laboratory of Antibody Engineering of Guangdong Higher Education Institutes, School of Laboratory Medicine and Biotechnology, Southern Medical University, Guangzhou, China; ^2^ Obstetrics and Gynecology Center, Nanfang Hospital, Guangzhou, China

**Keywords:** mRNA vaccine, molecular design, drug delivery, administration, COVID-19 mRNA vaccine

In the published article, there was a mistake in the order of the listed authors. The correct authors list appears above.

The authors apologize for this error and state that this does not change the scientific conclusions of the article in any way. The original article has been updated.

